# Burnout, Covert Narcissism, and Personality Traits: The Need to Distinguish Empathy Domains in Medical Residents

**DOI:** 10.3390/jcm15030982

**Published:** 2026-01-26

**Authors:** Adelina Alcorta-Garza, Oscar Vidal-Gutiérrez, Javier Alejandro Martínez-Moyano, Celia Beatriz González-Alcorta, Fernando Alcorta-Núñez, Mónica Lizeth Garza-García, Paola Azucena López-Sierra, Itzel Lidey Galaviz-Reynoso, Aminta Mariel Cortés-Almazán, Camila Alejandra Martínez-Roque, Juan Francisco González-Guerrero

**Affiliations:** 1 Coordination of Psycho-Oncology, Liaison Medicine and Palliative Care, Oncology Service, “José E. González” University Hospital, University Center Against Cancer, Universidad Autónoma de Nuevo León, Monterrey 64460, Mexico; ferchoalcorta9@gmail.com (F.A.-N.); monligza@gmail.com (M.L.G.-G.); pao_lpzz@hotmail.com (P.A.L.-S.); itzel.lidey@gmail.com (I.L.G.-R.); marielcortes00@gmail.com (A.M.C.-A.); camilamartinezroque@gmail.com (C.A.M.-R.); 2 Oncology Service, “José E. González” University Hospital, University Center Against Cancer, Universidad Autónoma de Nuevo León, Monterrey 64460, Mexico; vidal_oscar@hotmail.com (O.V.-G.); jvr_mm@hotmail.com (J.A.M.-M.); celiagzz@gmail.com (C.B.G.-A.); juanfglzg@gmail.com (J.F.G.-G.)

**Keywords:** burnout, covert narcissism, personality, empathy, medical residents

## Abstract

**Background/Objectives**: Identifying consistent patterns across empathy domains can help clinicians understand how empathy relates to burnout, covert narcissism, and other personality traits, thereby enhancing the effectiveness of clinical training. We examined empathy and assessed whether burnout, covert narcissism, and other personality traits show consistent associations across empathy domains. **Methods**: This cross-sectional study included 213 medical residents from a teaching and public tertiary care facility in Mexico. The Jefferson Scale of Empathy, the Maslach Burnout Inventory, the Hypersensitive Narcissism Scale, and the Zuckerman–Kuhlman Personality Questionnaire were applied. Nonparametric partial correlations were calculated, controlling for sex, age, specialty, year of residency, and psychological well-being. **Results**: On a 7-point Likert scale, the mean scores for perspective-taking, compassionate care, and the ability to stand in the patient’s shoes were 6.0 ± 0.8, 6.0 ± 1.0, and 4.1 ± 1.2, respectively. Depersonalization was negatively correlated with all empathy domains: perspective-taking (Spearman’s ρ = −0.20, *p* = 0.04), compassionate care (Spearman’s ρ = −0.30, *p* < 0.0001), and the ability to stand in the patient’s shoes (Spearman’s ρ = −0.25, *p* < 0.0001). The associations between other components of burnout, covert narcissism, and the remaining personality traits varied according to the domain of empathy. **Conclusions**: Depersonalization showed consistent, albeit modest, negative associations with all empathy domains, whereas the remaining psychological factors showed domain-specific relationships. Differentiating between empathy domains is essential, as it allows medical educators and clinicians to tailor interventions to specific components rather than treating empathy as a unitary construct.

## 1. Introduction

Empathy plays a central role in medical training programs worldwide. It is crucial from the start of medical education and becomes increasingly important throughout medical residents’ clinical training and practice. Educators, professional organizations, and research studies increasingly recognize empathy as a fundamental competency that directly affects patient care, clinical outcomes, and the development of future physicians. In Latin America, empathy has gained increasing attention as an essential component of medical professionalism and humanistic care, given the region’s social, cultural, and health system challenges. At the national level in Mexico, medical education occurs within a complex healthcare environment characterized by high patient volumes, resource constraints, and significant social disparities. In this context, it is very relevant for delivering patient-centered care and maintaining trust in the physician–patient relationship. Empathy is the ability of healthcare professionals to comprehend and respond compassionately to their patients’ emotions and needs. There are three key dimensions: perspective-taking, compassionate care, and the ability to stand in the patient’s shoes [1]. The construct contains two distinct but interconnected aspects: cognitive and affective. The cognitive element involves understanding the other person’s feelings or thoughts, and the affective aspect consists of sharing the other person’s emotional state. Both processes must function correctly [2,3]. On the other hand, burnout is characterized by emotional exhaustion, detachment, and a diminished sense of accomplishment, which can impair empathy [4]. Passalacqua et al. [5] investigated the longitudinal impact of burnout on empathy decline. They found that as stress levels increased, burnout also rose, and empathy decreased, ultimately affecting patient-centered communication practices. Personality is another psychological factor that affects the quality of the doctor–patient relationship in different ways. Extraversion or neuroticism influences how people cope with stress and interact with others. Sociability enhances interpersonal relationships and empathy, whereas the opposite is true for aggression and hostility [6]. Narcissism, in particular, is often characterized by its overtly grandiose traits, including thoughts of unlimited power and success, as well as an excessive need for praise and special treatment. However, there is also a lesser-known form called vulnerable narcissism, which is referred to as covert narcissism. These variants differ in self-regulatory functioning and may relate to empathy in distinct ways [7,8,9]. Covert narcissism has been specifically associated with lower levels of empathy [8,9]. It is characterized by hypersensitivity to criticism and fragile self-esteem, which may lead individuals to experience heightened emotional reactivity and distress in response to negative evaluations [10]. These characteristics are especially relevant in the context of medical residency because residents are repeatedly exposed to performance feedback and corrective evaluation while occupying subordinate roles.

Burnout, covert narcissism, and other personality traits might influence affective and cognitive domains of empathy differently. Burnout—mainly emotional exhaustion and depersonalization—is theorized to primarily affect the affective and relational domains of empathy. Stress and resource-depletion models posit that sustained emotional demands reduce clinicians’ capacity for emotional engagement, promoting detachment as a coping strategy. This process may selectively diminish empathic concern and patient-centered attitudes while leaving cognitive understanding relatively preserved. In contrast, covert narcissism features may more strongly disrupt the cognitive and motivational aspects of empathy, such as perspective-taking and adopting the patient’s viewpoint, due to competing internal demands and reduced outward attention allocation. Taken together, this framework supports the hypothesis that burnout, covert narcissism, and related personality traits exert domain-specific effects on empathy, rather than a global reduction. Examining empathy at the domain level, therefore, allows for a more precise understanding of how individual psychological factors may shape empathic functioning in medical trainees.

Medical educators should be alert to factors associated with empathy. Research on healthcare workers, including medical residents, has examined the relationship between different components of burnout and empathy. However, findings are inconsistent across studies, with reported associations varying by empathy domain, psychological construct assessed, and study population. Zakerkish et al. [11] reported significant correlations among perspective-taking, compassionate care, and the ability to stand in the patient’s shoes, suggesting partial convergence across empathy domains. In contrast, other studies report more selective or domain-specific associations. For example, Lamothe et al. [12] found that only emotional exhaustion was negatively associated with a single empathy domain—the ability to stand in the patient’s shoes—while no significant relationships were observed for other domains. Similarly, Tei et al. [13] reported an association between emotional exhaustion and perspective-taking; however, contrary to theoretical expectations, this relationship was positive. Personality-related findings also show domain-specific patterns. Agreeableness has been consistently associated with higher empathic concern and perspective-taking. Available research on covert narcissism has been conducted primarily among medical students. It has relied on global empathy scores without distinguishing among empathy domains [14], leaving a gap in understanding its manifestations during medical residency. Unlike medical students, residents must learn and provide patient care simultaneously, undergoing constant evaluation. This makes residency a crucial yet understudied context for identifying the relationship between vulnerable narcissism and empathy. Identifying consistent patterns across empathy domains can help clinicians understand how empathy relates to burnout, covert narcissism, and other personality traits—factors that may directly influence how medical residents interact with patients and colleagues. These insights can inform curriculum design by guiding interventions tailored to the specific factors associated with each empathy domain, thereby enhancing the effectiveness of clinical training.

This study aimed to examine empathy levels and assess whether burnout, covert narcissism, and other personality traits show consistent associations across empathy domains among medical residents. We hypothesized that emotional exhaustion, depersonalization, covert narcissism, and less adaptive personality traits (impulsive sensation seeking, neuroticism-anxiety, and aggression-hostility) would negatively correlate with empathy. Conversely, personal accomplishment and more adaptive personality traits (activity and sociability) would positively correlate with empathy.

## 2. Materials and Methods

This cross-sectional study was conducted between 2023 and 2024 at a public tertiary care teaching hospital. All medical residents enrolled in clinical training during the study period were eligible to participate, regardless of specialty or year of training. Participants were identified through the academic residency registry and invited by email (*n* = 276). The invitation, sent by the research team in coordination with residency program authorities, included a brief description of the study objectives, the voluntary nature of participation, and a secure link to an anonymous online questionnaire hosted on SurveyMonkey. A QR code was shared through WhatsApp for access. Residents were informed that participation or non-participation would not affect their academic evaluation or clinical responsibilities. No incentives were offered. We obtained a sample size of 213 participants, achieving a response rate of 77.1%. This size allowed us to detect effect sizes of 0.17 or greater with a one-tailed test at α = 0.05 and a power of 0.80 [15]. A one-tailed assumption was justified by a priori directional hypotheses grounded in theory and prior empirical evidence [4,5,6,8,9]. The protocol was approved by the Ethics, Biosafety, and Research Committees of the Dr. José Eleuterio González Hospital of the Autonomous University of Nuevo León, Mexico (code ON-2300003). Informed consent was obtained from all subjects involved in the study.

### 2.1. Study Variables

#### 2.1.1. Empathy

The Jefferson Scale of Physician Empathy for Physicians and Health Professionals was originally developed by Hojat et al. (2002) [1]. However, we chose the Spanish adaptation because we observed differences in item factor loadings between the Spanish and English versions, consistent with findings from a previous validation study among Mexican medical students [16]. The item “I do not allow myself to be touched by intense emotional relationships among my patients and their family members” loaded on the ability to stand in the patient’s shoes domain (factor coefficient = 0.50) rather than on the compassionate care domain (factor coefficient = −0.06). The adapted scale consists of three dimensions: perspective-taking (10 items; Cronbach’s Alpha = 0.85), compassionate care (7 items; Cronbach’s Alpha = 0.73), and the ability to stand in the patient’s shoes (3 items; Cronbach’s Alpha = 0.48). Total scale Cronbach’s α was 0.83 (20 items). Questions were answered on a 7-point Likert-type scale. Higher scores indicated greater empathy.

#### 2.1.2. Burnout

We used the Maslach Burnout Inventory developed by Maslach and Jackson (1981) [17]. The Spanish version of the Maslach Burnout Inventory has been validated among Mexican health personnel, demonstrating acceptable internal consistency and factorial validity [18,19]. It consists of 3 subscales: emotional exhaustion (9 items; Cronbach’s Alpha = 0.91), depersonalization (5 items; Cronbach’s Alpha = 0.81), and personal accomplishment (8 items; Cronbach’s Alpha = 0.73). Total scale Cronbach’s α was 0.90 (22 items). Questions were answered on a 6-point Likert-type scale. Higher scores indicated greater emotional exhaustion, depersonalization, and personal accomplishment. Burnout prevalence was estimated using cut-off scores established in studies of medical residents from other countries [20,21,22], as population-specific thresholds for Mexican residents were not available: ≥26 for high emotional exhaustion, ≥9 for high depersonalization, and ≥34 for high personal accomplishment. Although this approach facilitates comparability with the international literature, the results should be interpreted with caution, as cultural and contextual factors may influence score distributions.

#### 2.1.3. Covert Narcissism

We used the Hypersensitive Narcissism Scale (HSNS) developed by Hendin and Cheek (1997) [23]. A Spanish version of the instrument has been previously validated in a clinical sample in Spain [24]. However, given linguistic and cultural differences, we directly adapted the original instrument, using an independent translation to ensure conceptual and cultural equivalence within the Mexican healthcare context. It consists of one dimension. Respondents rated on a 5-point Likert-type scale statements related to how easily they felt hurt by others, how they sought validation, or how they reacted when they did not receive the attention they thought they deserved (10 items, Cronbach’s Alpha = 0.80). Higher scores indicated a higher tendency towards a covert narcissistic personality trait.

#### 2.1.4. Personality

We used the Zuckerman–Kuhlman personality questionnaire (ZKPQ) to assess five basic personality traits (1993) [25]. The ZKPQ has been validated in Spain [26]. However, given linguistic and cultural differences, we directly adapted the original instrument using an independent translation to ensure conceptual and cultural equivalence in the Mexican population. The scale comprises five dimensions: Impulsive Sensation Seeking (19 items, Kuder–Richardson = 0.79), Neuroticism–Anxiety (19 items, Kuder–Richardson = 0.89), Aggression–Hostility (17 items, Kuder–Richardson = 0.62), Sociability (17 items, Kuder–Richardson = 0.73), and Activity (17 items, Kuder–Richardson = 0.64). Participants indicated whether they agreed (1 point) or disagreed (0 points) with each item. Higher scores indicated greater levels of impulsive sensation seeking, neuroticism–anxiety, aggression–hostility, sociability, and activity.

#### 2.1.5. Other Variables

Psychological well-being was assessed using the General Health Questionnaire (GHQ-12) (Goldberg et al. 1997) [27] (12 items; Cronbach’s Alpha = 0.52). Higher scores indicated greater psychological distress. GHQ-12 has demonstrated acceptable internal consistency and factorial validity in the general adult population in Spain [28]. However, given linguistic and cultural differences, we directly adapted the original instrument using an independent translation to ensure conceptual and cultural equivalence in the Mexican population. Sociodemographic factors and degree-related information (specialty and year of residence) were also collected. Specialties were categorized into four groups, following the classification proposed by Hojat et al. [6]: 1. Primary Care Specialties: These are people-oriented and primarily office-based. They provide initial health and illness assessments, as well as comprehensive episodic and long-term care for a wide range of medical conditions (e.g., family medicine). 2. Non-Primary Care Specialties: These involve a mix of ambulatory and hospital-based practice. They provide episodic or long-term care for specific medical conditions (e.g., endocrinology). 3. Technology-Oriented Specialties: These are primarily hospital-based, with some office-based activities. They focus on highly skilled and specialized therapeutic techniques or procedures (e.g., general surgery). 4. Procedure-Oriented Hospital-Based Specialties: These primarily provide specialized diagnostic procedures or conduct applied laboratory research. Professional interactions are mainly with colleagues rather than patients (e.g., nuclear medicine).

### 2.2. Statistical Analysis

Data were screened before analysis to identify missing and atypical values. Cases with incomplete responses on the study variables were excluded from the corresponding analyses (listwise deletion). Descriptive statistics, including means and standard deviations, were utilized for continuous variables, while frequency distributions were used for categorical variables. Zero-order Spearman correlation coefficients were estimated for burnout, covert narcissism, other personality traits, and empathy, given non-normal data distributions (K-S < 0.05). In a subsequent analysis, nonparametric partial correlations were estimated while controlling for potential confounding variables (sex, age, specialty, year of residence, and psychological well-being). To minimize Type I error due to multiple comparisons, a Bonferroni correction was applied. All reported *p*-values for zero-order and partial correlation analyses were one-tailed, reflecting directional hypotheses specified before analysis. We conducted a sensitivity analysis given the low Cronbach’s α of 0.48 for the ability to stand in the patient’s shoes domain. Excluding the relocated item increased Cronbach’s α to 0.63. Nonetheless, the pattern of results remained similar (see Supplementary Table S1). Statistical analyses were performed using IBM SPSS Statistics (IBM Corp., Armonk, NY, USA, Version 22).

## 3. Results

The mean age was 28.2 ± 3.0; 54.9% were female, and 88.7% were married. Specialty groups were as follows: non-primary care (40.4%), primary care (26.8%), technology (19.2%), and procedure-oriented (13.6%). The first-year residency was the most prevalent (86.9%), followed by the second-year (7.5%) and third-year or higher (5.6%). The mean overall empathy score was 115.0 ± 14.3, corresponding to 5.8 ± 0.7 on a 7-point Likert scale. The empathy domain with the lowest outcome was the ability to stand in the patient’s shoes (4.1 ± 1.2 on the Likert scale). Perspective-taking and compassionate care mean scores were 6.0 ± 0.8 and 6.0 ± 1.0 on the 7-point Likert scale, respectively. Regarding burnout, the prevalence of high emotional exhaustion, depersonalization, and personal accomplishment was 52.1%, 36.6%, and 77.9%, respectively. Covert narcissism and most personality traits were below the midpoint of the expected score range (Table 1).

Women scored higher in the ability to stand in the patient’s shoes domain (4.4 ± 1.2 vs. 3.8 ± 1.2 on a 7-point Likert scale, *p* < 0.0001); no significant sex differences were found in the other domains. Age was not significantly correlated with any of the empathy domains, nor specialty or year of residency. Table 2 presents zero-order and partial correlations between burnout dimensions, personality traits, and empathy domains. Overall, statistically significant adjusted associations were small to moderate in magnitude (ρ ≈ 0.19–0.35). Depersonalization showed the most consistent pattern of associations across empathy domains, with effect sizes ranging from small to moderate. It was associated with lower perspective-taking (ρ = −0.20, *p* = 0.04), lower compassionate care (ρ = −0.30, *p* < 0.0001), and reduced ability to stand in the patient’s shoes (ρ = −0.25, *p* < 0.0001). Covert narcissism showed small but consistent negative associations with two empathy domains, perspective-taking (ρ = −0.19, *p* = 0.05) and compassionate care ρ = −0.21, *p* = 0.02). The remaining personality traits showed limited, domain-specific associations.

## 4. Discussion

The study population consisted of young medical residents, primarily in their first year of residency. Our first objective focused on assessing levels of burnout, covert narcissism, other personality traits, and empathy. We found that the mean overall empathy score was high. The domain breakdown revealed high levels of perspective-taking and compassionate care, along with a low-to-moderate ability to stand in the patient’s shoes. Other studies in medical residents show comparable results [11,22,29,30]. Medical residents often encounter stressful situations during their training, particularly in clinical settings. Residency typically involves long hours, a high patient volume, and limited sleep [31]. In our sample, over half of the participants reported high emotional exhaustion, approximately 40% exhibited high depersonalization, and around 80% had a high sense of personal accomplishment. A study conducted in another Latin American country, Brazil, found different patterns, lower emotional exhaustion (44.8%), higher depersonalization (64.2%), and lower personal accomplishment (52.2%) [20]. Residency represents a formative stage of professional development during which cultural and organizational contexts may substantially shape empathy, burnout, and coping patterns. In Mexico, clinical training occurs within a sociocultural environment characterized by strong interpersonal values, respect for authority, and hierarchical relationships in healthcare settings. These features may influence how empathy is expressed, perceived, and reported, particularly in formal clinical interactions. Examining empathy and burnout among residents, therefore, provides critical insight into how these context-dependent factors interact early in physicians’ careers, with potential long-term implications for professional identity formation, well-being, and patient care quality.

Empathy is not a single construct; it encompasses at least two distinct domains: one affective and the other cognitive. We considered that burnout dimensions and personality traits may be linked to these components differently. Therefore, our second objective was to investigate whether the associations were consistent across the various empathy domains. All associations have minor to moderate strength. We found that depersonalization was the only factor consistently associated across all empathy domains, different from Zakerkish et al. [11], who found that all three burnout components were related to all three empathy domains. Emotional exhaustion was associated with two domains: compassionate care and the ability to stand in the patient’s shoes. In comparison, Lamothe et al. [12] found it correlated only with the ability to stand in the patient’s shoes. Additionally, we did not find emotional exhaustion associated with perspective-taking, in contrast to the findings of Tei et al. [13], who reported that this was the only component significantly associated with it. These discrepancies may be attributed to differences in cultural context, sample characteristics such as the medical field, specialty distribution, or stage of training. The study by Zakerkish et al. [11] was conducted among medical residents in Iran. In contrast, the studies by Lamothe et al. [12] and Tei et al. [13] were conducted among general practitioners in France and nurses in Japan, respectively. We identified low covert narcissism and low to moderate impulsive sensation-seeking, neuroticism-anxiety, aggression-hostility, sociability, and activity. Covert narcissism and neuroticism-anxiety were the only personality traits negatively associated with compassionate care, with small to moderate effect sizes. Among the major personality traits, only agreeableness showed a significant correlation with empathic concern and perspective-taking among pediatric residents [32]. Our findings revealed statistically significant associations, albeit modest in size. From an intervention perspective, these findings could still be meaningful as they identify modifiable targets. Recognition of domain-specific patterns and associated psychological factors enables medical educators to target educational strategies. For instance, initiatives to improve perspective-taking may focus on reflective listening and understanding patients’ reasoning while addressing emotional exhaustion and depersonalization. In contrast, compassionate care may be better addressed through experiential learning approaches, considering the influence of the resident’s covert narcissism and the neuroticism-anxiety personality trait for achieving a better outcome.

In this study, women scored higher than men only in the ability to stand in the patient’s shoes domain. This result aligns with cross-cultural findings showing that women tend to be more emotionally responsive and sensitive to patients’ experiences [30,33]. In the clinical context, this female advantage may significantly enhance their ability to connect with patients, fostering stronger relationships. Moreover, the pattern suggests that sex differences in empathy among medical residents are domain-specific rather than global. The absence of sex differences in perspective-taking and compassionate care indicates substantial overlap between men and women across core components of empathy. From an educational perspective, we believe these findings do not support sex-differentiated empathy training; instead, they underscore the importance of fostering empathic engagement across all learners while remaining sensitive to individual and contextual variability. There were no differences in empathy by residency year or specialty type, contrary to what other authors have reported [6,30,33]. The high proportion of first-year residents likely limited variability across training stages, potentially reducing the ability to detect year-related differences. Additionally, all participants were drawn from a single public tertiary care hospital, where shared institutional culture, workload, and clinical demands may exert a homogenizing effect across specialties, reducing the ability to detect differences.

### Study Limitations

The study population comprised medical residents from a single public tertiary care hospital, predominantly in their first year of training, which may limit the generalizability of the findings. Senior residents may differ in their levels of empathy and coping strategies, and patient complexity may also influence residents’ experiences. Furthermore, organizational and cultural norms in public healthcare may differ from those in private institutions, so the results may not apply to residents in private hospitals or in other contexts. We recognized two other potential biases. Because all measures relied on self-report instruments, response bias is possible; despite anonymity and the use of validated tools, participants may have overreported socially desirable qualities, such as empathy, and underreported less favorable traits, such as narcissistic tendencies. Also, because participation was voluntary, selection bias cannot be ruled out. Individuals with higher empathic engagement may have been more inclined to respond, potentially inflating or attenuating observed associations. As a result, the findings should be interpreted with appropriate caution. The study design was cross-sectional, limiting causal inference for each examined relationship. Understanding the complex links between burnout and empathy requires a longitudinal approach to assess whether burnout affects empathy or vice versa. Finally, we acknowledge concern about the Cronbach’s α of the psychological well-being scale, which fell below the conventional acceptability threshold. This measure was used as a covariate in the partial correlation analyses because of its theoretical relevance as a potential confounder. However, the limited reliability would be expected to attenuate observed associations, yielding more conservative estimates rather than inflated effect sizes. Similarly, although Cronbach’s α for the ‘ability to stand in the patient’s shoes’ domain was low, sensitivity analyses supported the stability of the observed associations despite the scale’s reduced reliability.

## 5. Conclusions

The findings highlight the need to address empathy domains, as the associations between burnout components, covert narcissism, and other personality traits varied across different aspects of empathy. Depersonalization was associated with all empathy domains, followed by emotional exhaustion and personal accomplishment, which were associated with two domains each, and covert narcissism and neuroticism-anxiety personality traits, which were associated with a single domain each. Studies such as this enable medical educators and clinicians to focus on psychological factors most closely linked to specific empathy domains that require improvement. Future research should examine the efficacy of domain-specific empathy training interventions while considering personality traits and burnout risk, using longitudinal and experimental designs, particularly during residency. Additionally, they could clarify how personality traits and burnout-related factors influence responsiveness to empathy training over time. Comparative research across institutions and cultural contexts may further help determine the generalizability of domain-based approaches and inform the personalization of empathy-training initiatives.

## Figures and Tables

**Table 1:** Descriptive statistics of empathy and psychological factors.

	Mean ± SD	Median	Expected Score Range	Observed Score Range
Empathy				
Perspective-taking ^a^	60.4 ± 8.5	62	10–70	10–70
Compassionate care ^a^	42.3 ± 6.7	44	7–49	15–49
Ability to stand in the patient’s shoes ^a^	12.3 ± 3.7	12	3–21	4–21
Total ^a^	115.0 ± 14.3	117	20–140	79–140
Burnout				
Emotional exhaustion ^b^	26.4 ± 12.6	27	0–54	0–54
Depersonalization ^b^	7.6 ± 6.3	6	0–30	0–28
Personal accomplishment ^c^	38.2 ± 6.7	40	0–48	13–48
Narcissism (covert) ^b^	22.3 ± 7.6	22	10–50	10–41
Psychological well-being ^b^	4.4 ± 2.3	4	0–12	0–12
Personality trait				
Impulsive sensation seeking ^b^	6.8 ± 3.9	6	0–17	0–18
Neuroticism-anxiety ^b^	7.6 ± 5.2	7	0–19	0–19
Aggression-hostility ^b^	4.8 ± 2.6	5	0–17	0–11
Activity ^c^	8.0 ± 3.1	8	0–17	0–16
Sociability ^c^	6.5 ± 3.3	6	0–17	0–15

^a^ The higher the score, the greater the empathy. ^b^ The higher the score, the higher the emotional exhaustion and depersonalization, the greater the psychological distress, or the less adaptive the personality trait. ^c^ The higher the score, the higher the personal accomplishment (lower burnout), or the more adaptive the personality trait.

**Table 2:** Spearman’s rho correlation coefficient between psychological factors and empathy domains.

	Empathy Domain					
	PT	CC	A	PT	CC	A
Psychological factor	Zero-order ρ			Partial ρ ^d^		
Burnout						
Emotional exhaustion ^a^	−0.11	−0.19 *	−0.25 **	−0.13	−0.21 *	−0.27 **
Depersonalization ^a^	−0.19 *	−0.28 **	−0.29 **	−0.20 *	−0.30 **	−0.25 **
Personal accomplishment ^a^	0.28 **	0.34 **	0.20 *	0.28 **	0.35 **	0.17
Covert narcissism ^b^	−0.18	−0.20 *	−0.19 *	−0.19 *	−0.21 *	−0.18
Other personality traits						
Impulsive sensation-seeking ^c^	0.02	−0.17	−0.21 *	0.03	−0.16	−0.14
Neuroticism-anxiety ^c^	−0.02	−0.16	−0.09	−0.01	−0.21 *	−0.15
Aggression-hostility ^c^	−0.14	−0.18	−0.12	−0.14	−0.18	−0.08
Activity ^c^	0.02	0.00	−0.04	−0.01	0.00	−0.04
Sociability ^c^	0.06	0.09	0.05	0.06	0.11	0.10

PT: Perspective taking, CC: Compassionate care, A: Ability to stand in the patient’s shoes. ^a^ Higher scores indicated greater emotional exhaustion, depersonalization, and personal accomplishment. ^b^ Higher scores indicated a higher tendency towards a covert narcissistic personality trait. ^c^ Higher scores indicated greater levels of impulsive sensation seeking, neuroticism–anxiety, aggression–hostility, sociability, and activity. ^d^ Controlling for sex, age, specialty, year of residence, and psychological well-being. * *p* < 0.05 (one-sided), ** *p* < 0.01 (one-sided). Note: To control for multiple testing, a Bonferroni correction was applied for nine comparisons per empathy domain (emotional exhaustion, depersonalization, personal accomplishment, covert narcissism, impulsive sensation-seeking, neuroticism–anxiety, aggression–hostility, activity, and sociability).

## Data Availability

The datasets used and/or analyzed in the current study are available from the corresponding author upon reasonable request.

## Supplementary Materials

The following supporting information can be downloaded at: https://www.mdpi.com/article/10.3390/jcm15030982/s1, Table S1: Sensitivity analysis on the Ability to stand in the patient’s shoes domain.
