# Supplementary material for: Burnout, Covert Narcissism, and Personality Traits: The Need to Distinguish Empathy Domains in Medical Residents
Source: J Clin Med. 2026 Jan 26;15(3):982. doi: 10.3390/jcm15030982 (PMC12898612; doi:10.3390/jcm15030982)
Supplement: Supplementary file 1 [file jcm-15-00982-s001.zip › jcm-4055380-supplementary.pdf]

S1. Sensitivity analysis on the Ability to stand in the patient's shoes domain

	Empathy domain					
	Spanish version			English version		
	PT (10 items; Cronbach's α= 0.85)	CC (7 items; Cronbach's α = 0.73)	A (3 items; Cronbach's α = 0.48)	PT (10 items; Cronbach's α = 0.85)	CC (8 items; Cronbach's α = 0.68)	A (2 items; Cronbach's α = 0.63)
Psychological factor	Partial ρ [a]			Partial ρ [a]		
Burnout						
Emotional exhaustion	-0.13	**-0.21***	**-0.27****	-0.13	**-0.24****	**-0.27****
Depersonalization	**-0.20***	**-0.30****	**-0.25****	**-0.20***	**-0.30****	**-0.27****
Personal accomplishment	**0.28****	**0.35****	0.17	**0.28****	**0.31****	**0.25****
Covert narcissism	**-0.19***	**-0.21***	-0.18	**-0.19***	**-0.21***	**-0.22****
Other personality traits						
Impulsive sensation-seeking	0.03	-0.16	-0.14	0.03	-0.17	-0.14
Neuroticism-anxiety	-0.01	**-0.21***	-0.15	-0.01	**-0.22***	-0.13
Aggression-hostility	-0.14	-0.18	-0.08	-0.14	-0.18	-0.09
Activity	-0.01	0.00	-0.04	-0.01	0.00	-0.09
Sociability	0.06	0.11	0.10	0.06	0.10	0.09

PT: Perspective taking, CC: Compassionate care, A: Ability to stand in the patient's shoes. [a] Controlling for sex, age, specialty, year of residence, and psychological well-being. *$p < 0.05$ (one-sided), ** $p < 0.01$ (one-sided). Bonferroni correction was applied for nine comparisons per empathy domain (emotional exhaustion, depersonalization, personal accomplishment, covert narcissism, impulsive sensation-seeking, neuroticism–anxiety, aggression–hostility, activity, and sociability).
